# Complete Genome Sequence of Lactobacillus johnsonii MR1, Isolated from a BALB/c Mouse Cecum

**DOI:** 10.1128/mra.01078-22

**Published:** 2022-12-13

**Authors:** Keerthikka Ravi, Marcus Rauch, Susan V. Lynch, Nicholas W. Lukacs, Gary B. Huffnagle

**Affiliations:** a Department of Molecular, Cellular, and Developmental Biology, University of Michigan, Ann Arbor, Michigan, USA; b Division of Gastroenterology, University of California, San Francisco, San Francisco, California, USA; c Division of Pulmonary and Critical Care Medicine, University of California, San Francisco, San Francisco, California, USA; d Mary H. Weiser Food Allergy Center, University of Michigan, Ann Arbor, Michigan, USA; e Department of Pathology, University of Michigan, Ann Arbor, Michigan, USA; University of Rochester School of Medicine and Dentistry

## Abstract

Lactobacillus johnsonii strain MR1, which was isolated from the cecum of a BALB/cJ mouse in an airway allergy model, can decrease allergic airway inflammation in the model upon oral administration. Long-read sequencing of this isolate, which was performed using a MinION sequencer, yielded a single, closed genome of 1,953,837 bp, with a GC content of 34.67%.

## ANNOUNCEMENT

Lactobacillus johnsonii, a lactic acid bacterium, is associated with a healthy gut microbiome. Classified as generally regarded as safe (GRAS) (1), this species has several health-promoting effects, including inhibition and exclusion of pathogens (2–4) and host immunomodulation (5, 6). L. johnsonii strain MR1, which was sequenced here, was isolated from the cecum of a BALB/cJ mouse (purchased from the Jackson Laboratory) in an airway allergy study performed at the University of Michigan, in which house-dust-mediated protection against disease was associated with significant enrichment of L. johnsonii in the cecum (7). Oral supplementation of mice with L. johnsonii MR1 during respiratory syncytial virus infection resulted in decreased airway inflammation and reduced allergic airway disease in both adults and neonates (5, 8). Here, we report the complete genome sequence of immunomodulatory L. johnsonii strain MR1.

L. johnsonii strain MR1 was initially isolated from the cecum of a mouse and identified as described by Fujimura et al. (7). A frozen stock of L. johnsonii strain MR1 was inoculated in de Man, Rogosa, and Sharpe (MRS) broth and grown overnight at 37°C under static aerobic conditions. High-molecular-weight (HMW) DNA for long-read sequencing was isolated from this overnight bacterial culture using the Nanobind HMW DNA extraction kit (Circulomics) with RNase treatment.

Sequencing libraries were prepared by using the ligation sequencing kit (SQK-LSK109) and selecting DNA fragments of all sizes with the short fragment buffer (SFB), according to the Oxford Nanopore Technologies library preparation protocol. The libraries were sequenced on an R9.4.1 flow cell for 3 h using a MinION sequencer (Oxford Nanopore Technologies). Base calling for the raw reads was performed using Guppy v4.3.4 in high-accuracy mode. A total of 86,118 reads, with an *N*_50_ value of 5,730 bp, were obtained after base calling. The genome sequences were assembled *de novo* using Flye v2.8.1 (9) with default parameters. The assembly was further polished through two rounds with Racon v1.4.20 (10) using the default settings for all parameters except the scores for matching and mismatching bases (–match 8 –mismatch -6). A final round of polishing was performed with Medaka v1.2.3 (11) using default parameters. MR1 (genome coverage, 120×) was assembled into one circular contig with a length of 1.9 Mb. Circularization of the contig was confirmed through visualization of the assembled sequence in Bandage (12).

The MR1 genome has a GC content of 34.67%. Sequence annotation for the strain was performed using the NCBI Prokaryotic Genome Annotation Pipeline (PGAP) (13). The annotation predicted 1,879 genes, 1,776 coding sequences, and 103 RNA genes (including 79 tRNAs, 21 rRNAs, and 3 noncoding RNAs).

### Data availability.

All data are available under BioProject accession number PRJNA791710 and BioSample accession number SAMN23978983. The genome was deposited under GenBank accession number CP091981.1. Nanopore base-called data were submitted to the SRA and are available under SRA accession number SRR18944281.

## References

[1] Vazquez-Munoz R, Thompson A, Russell JT, Sobue T, Zhou Y, Dongari-Bagtzoglou A. 2022. Insights from the *Lactobacillus johnsonii* genome suggest the production of metabolites with antibiofilm activity against the pathobiont *Candida albicans*. Front Microbiol 13:853762. doi:10.3389/fmicb.2022.853762. 35330775 PMC8940163

[2] He T, Zhu YH, Yu J, Xia B, Liu X, Yang GY, Su JH, Guo L, Wang ML, Wang JF. 2019. *Lactobacillus johnsonii* L531 reduces pathogen load and helps maintain short-chain fatty acid levels in the intestines of pigs challenged with *Salmonella enterica* Infantis. Vet Microbiol 230:187–194. doi:10.1016/j.vetmic.2019.02.003. 30827387

[3] Charlet R, Bortolus C, Sendid B, Jawhara S. 2020. *Bacteroides thetaiotaomicron* and *Lactobacillus johnsonii* modulate intestinal inflammation and eliminate fungi via enzymatic hydrolysis of the fungal cell wall. Sci Rep 10:11510. doi:10.1038/s41598-020-68214-9. 32661259 PMC7359362

[4] La Ragione RM, Narbad A, Gasson MJ, Woodward MJ. 2004. In vivo characterization of *Lactobacillus johnsonii* FI9785 for use as a defined competitive exclusion agent against bacterial pathogens in poultry. Lett Appl Microbiol 38:197–205. doi:10.1111/j.1472-765x.2004.01474.x. 14962040

[5] Fonseca W, Lucey K, Jang S, Fujimura KE, Rasky A, Ting HA, Petersen J, Johnson CC, Boushey HA, Zoratti E, Ownby DR, Levine AM, Bobbit KR, Lynch SV, Lukacs NW. 2017. *Lactobacillus johnsonii* supplementation attenuates respiratory viral infection via metabolic reprogramming and immune cell modulation. Mucosal Immunol 10:1569–1580. doi:10.1038/mi.2017.13. 28295020 PMC5599307

[6] Kingma SD, Li N, Sun F, Valladares RB, Neu J, Lorca GL. 2011. *Lactobacillus johnsonii* N6.2 stimulates the innate immune response through Toll-like receptor 9 in Caco-2 cells and increases intestinal crypt Paneth cell number in biobreeding diabetes-prone rats. J Nutr 141:1023–1028. doi:10.3945/jn.110.135517. 21490291

[7] Fujimura KE, Demoor T, Rauch M, Faruqi AA, Jang S, Johnson CC, Boushey HA, Zoratti E, Ownby D, Lukacs NW, Lynch SV. 2014. House dust exposure mediates gut microbiome *Lactobacillus* enrichment and airway immune defense against allergens and virus infection. Proc Natl Acad Sci USA 111:805–810. doi:10.1073/pnas.1310750111. 24344318 PMC3896155

[8] Fonseca W, Malinczak CA, Fujimura K, Li D, McCauley K, Li J, Best SKK, Zhu D, Rasky AJ, Johnson CC, Bermick J, Zoratti EM, Ownby D, Lynch SV, Lukacs NW, Ptaschinski C. 2021. Maternal gut microbiome regulates immunity to RSV infection in offspring. J Exp Med 218:e20210235. doi:10.1084/jem.20210235. 34613328 PMC8500238

[9] Kolmogorov M, Yuan J, Lin Y, Pevzner PA. 2019. Assembly of long, error-prone reads using repeat graphs. Nat Biotechnol 37:540–546. doi:10.1038/s41587-019-0072-8. 30936562

[10] Vaser R, Sović I, Nagarajan N, Šikić M. 2017. Fast and accurate de novo genome assembly from long uncorrected reads. Genome Res 27:737–746. doi:10.1101/gr.214270.116. 28100585 PMC5411768

[11] Oxford Nanopore Technologies. 2018. Medaka: sequence correction provided by ONT Research. https://github.com/nanoporetech/medaka.

[12] Wick RR, Schultz MB, Zobel J, Holt KE. 2015. Bandage: interactive visualization of de novo genome assemblies. Bioinformatics 31:3350–3352. doi:10.1093/bioinformatics/btv383. 26099265 PMC4595904

[13] Tatusova T, Dicuccio M, Badretdin A, Chetvernin V, Nawrocki EP, Zaslavsky L, Lomsadze A, Pruitt KD, Borodovsky M, Ostell J. 2016. NCBI Prokaryotic Genome Annotation Pipeline. Nucleic Acids Res 44:6614–6624. doi:10.1093/nar/gkw569. 27342282 PMC5001611

